# [Corrigendum] Targeting ribonucleotide reductase M2 subunit by small interfering RNA exerts anti-oncogenic effects in gastric adenocarcinoma 

**DOI:** 10.3892/or.2026.9183

**Published:** 2026-08-20

**Authors:** Wei Kang, Joanna H.M. Tong, Anthony W.H. Chan, Junhong Zhao, Shiyan Wang, Yujuan Dong, Frankie M.C. Sin, Saifung Yeung, Alfred S.L. Cheng, Jun Yu, Kafai To

Oncol Rep 31: 2579–2586, 2014; DOI: 10.3892/or.2014.3148

Subsequently to the publication of the above article, an interested reader drew to the Editor's attention that apparently the same set of GADPH data panels had been featured in a number of research articles by this group, even though it appeared as if these were intended to have shown the same experimental conditions in most cases. The authors have realized that the GAPDH loading control panel in question, shown in Fig. 1C on p. 2581, had been inappropriately used to represent the same batch of tissue samples in several articles. Although the tissue samples were from the same batch, the authors recognize that using the same GAPDH panel as the loading control across a number of different publications was inappropriate. To address this issue, the authors have replaced both the RRM2 and the GAPDH data in Fig. 1C of the above paper, and the revised version of Fig. 1 is shown on the next page.

In addition, upon re-examining their original data, the authors also realized that certain images captured for the invasion assay in Fig. 3C on p. 2584 contained overlapping sections (for example, the ‘Scramble siRNA’ and ‘RPM2 siRNA’ data panels for the AGS cell line). Therefore, the invasion assay experiments were also repeated, and the corresponding panels in Fig. 3C have been replaced. The revised version of Fig. 3 is shown on the second subsequent page. The authors wish to emphasize that the errors made in assembling the data in these figures did not affect the overall conclusions reported in the paper. The authors are grateful to the Editor of *Oncology Reports* for granting them this opportunity to publish a Corrigendum, and apologize to both the Editor and the readership for any inconvenience caused.

## Figures and Tables

**Figure 1.**
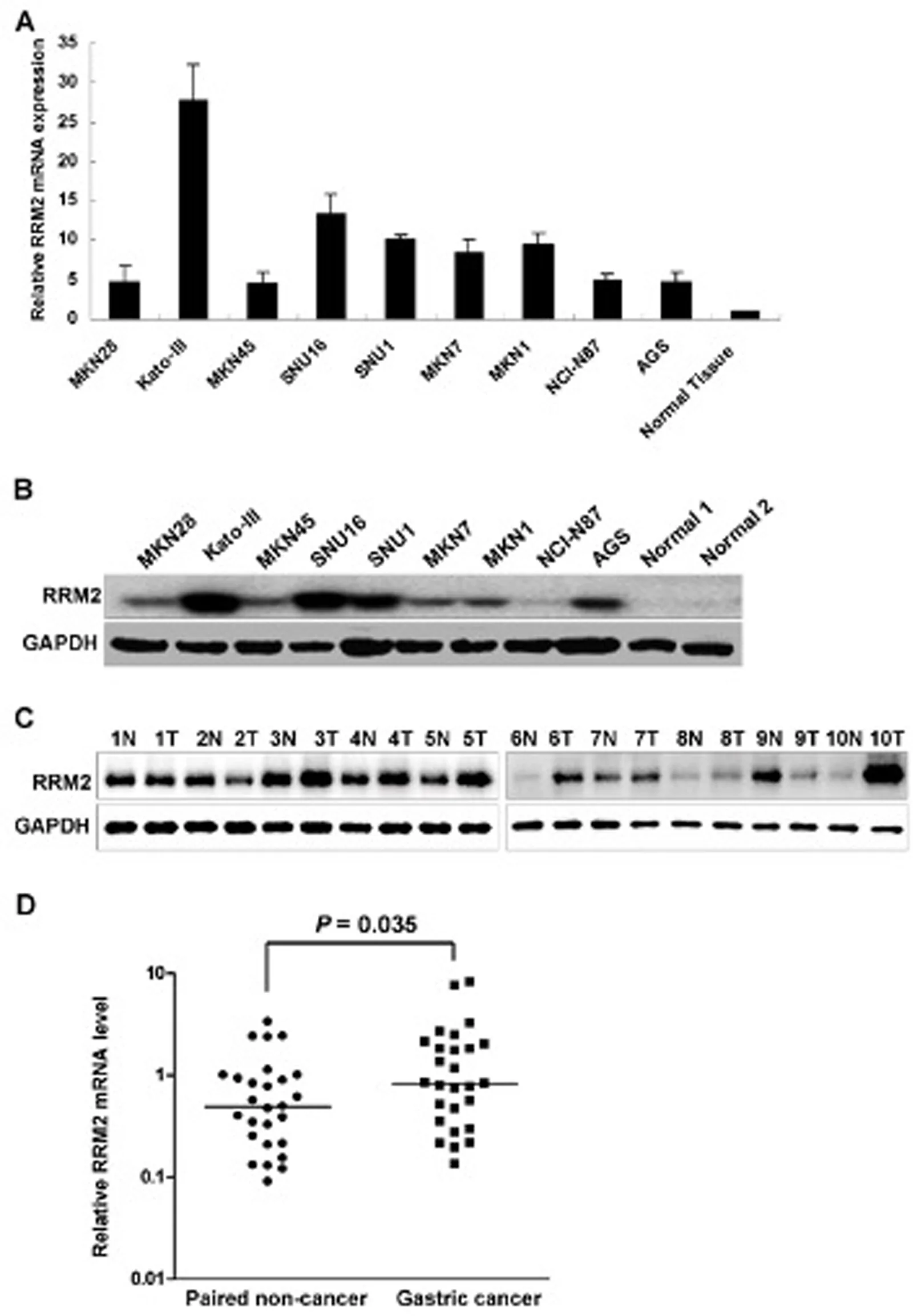
RRM2 upregulation in gastric cancer cell lines and primary gastric tumors. (A) RRM2 mRNA expression in 9 gastric cancer cell lines compared with normal gastric mRNA commercially available from Ambion (AM 7996). The experiment was performed in triplicates. (B) RRM2 protein expression in gastric cancer cell lines and 2 normal gastric mucosal tissues from patients with weight reduction gastric surgery. (C) Western blotting of RRM2 in 10 pairs of gastric tumors (T) and the corresponding non-tumorous mucosa (N). (D) Dot plot of qRT-PCR results from RRM2 mRNA expression level in 27 pairs of gastric adenocarcinoma and the corresponding non-tumorous mucosa (P=0.035). RRM2, ribonucleotide reductase M2 subunit.

**Figure 3.**
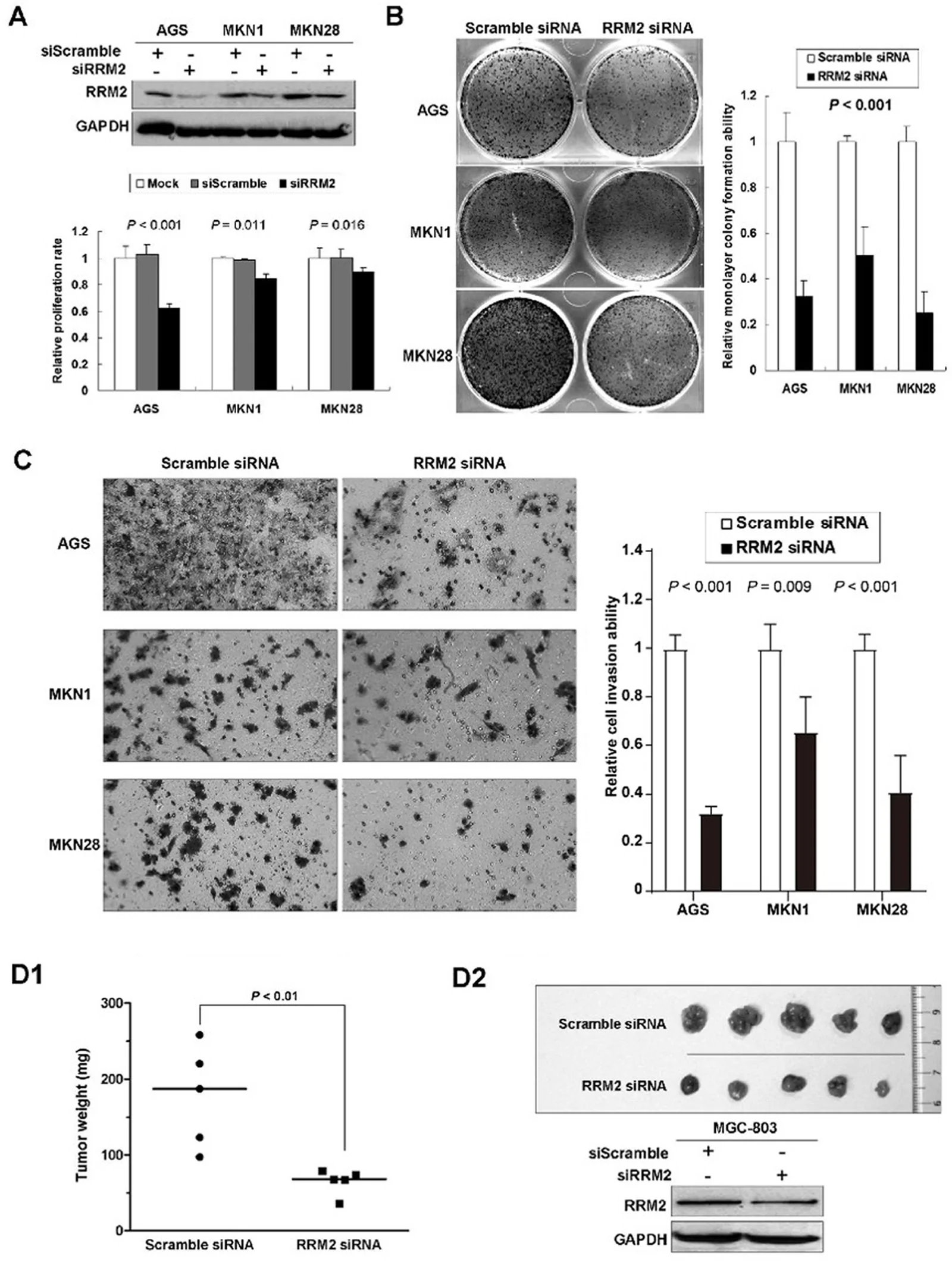
RRM2 knockdown by siRNA in gastric cancer cell lines. (A) Transfection with RRM2 siRNA reduced RRM2 protein expression in AGS, MKN1 and MKN28 cells. Five-day MTT assays revealed RRM2 siRNA suppressed gastric cancer cell proliferation. (B) siRRM2 reduced monolayer colony formation to 32.4, 50.2 and 25.1% in AGS, MKN1 and MKN28 cells, respectively. The experiments were performed in triplicates. (C) Representative images of invaded cells are shown. The number of invaded cells was reduced in siRRM2 transfectants compared to scramble controls in AGS, MKN1 and MKN28 cells. The number of cells was counted in three random microscopic fields and the error bars represented standard deviations. (D1) MGC-803-siRRM2 formed smaller xenograft tumors than MGC-803-si-scramble group 3 weeks after inoculation. (D2) Upper, xenograft images; lower, western blotting validation of RRM2 expression in the xenografts. RRM2, ribonucleotide reductase M2 subunit.

